# Supplementary material for: Decrease in the usual walking speed and body fat percentage associated with a deterioration in long-term care insurance certification levels
Source: PeerJ. 2024 Jun 21;12:e17529. doi: 10.7717/peerj.17529 (PMC11195544; doi:10.7717/peerj.17529)
Supplement: Supplemental Information 1 [file peerj-12-17529-s001.docx]

**Table S1. Details of the participants’ diseases**

|  | Number |
| --- | --- |
| Cerebrovascular disease | 48 |
| Orthopedic disease | 54 |
| Cancer | 15 |
| Intractable neurological disease | 13 |
| Circulatory disease | 23 |
| Respiratory disease | 13 |
| Diabetes | 23 |
| Hypertension | 44 |
| Osteoporosis | 11 |
