# Supplementary material for: Decrease in the usual walking speed and body fat percentage associated with a deterioration in long-term care insurance certification levels
Source: PeerJ. 2024 Jun 21;12:e17529. doi: 10.7717/peerj.17529 (PMC11195544; doi:10.7717/peerj.17529)
Supplement: Supplemental Information 3 — The analysis was conducted after excluding the Improvement group ( n = 12). Dependent variable: Maintenance group = 0; Deterioration group = 1. Change in body fat percentage: body fat percentage at follow-up minus the body fat percentage at baseline. Model I: Non-adjusted. Model II: Adjusted for sex, LTCI certification level, and intractable neurological disease. CI, confidence interval; LTCI, long-term care insurance. [file peerj-12-17529-s003.docx]

**Table S3. Association between certification level deterioration and changes in body fat percentage using binomial logistic regression analysis**

|  | β | Odds ratio | 95% CI | *P*-value |
| --- | --- | --- | --- | --- |
| Model Ⅰ | | | | |
| Change in body fat percentage | -0.247 | 0.781 | 0.645–0.947 | **0.012** |
| Model Ⅱ | | | | |
| Change in body fat percentage | -0.264 | 0.768 | 0.629–0.938 | **0.010** |

The analysis was conducted after excluding the Improvement group (n=12).

Dependent variable: Maintenance group=0; Deterioration group=1.

Change in body fat percentage: body fat percentage at follow-up minus the body fat percentage at baseline.

Model Ⅰ: Non-adjusted.

Model II: Adjusted for sex, LTCI certification level, and intractable neurological disease.

CI, confidence interval; LTCI, long-term care insurance.
